# Altered RNome expression in Murine Gastrocnemius Muscle following Exposure to Jararhagin, a Metalloproteinase from *Bothrops jararaca* Venom

**DOI:** 10.3390/toxins14070472

**Published:** 2022-07-09

**Authors:** Andrezza Nascimento, Bianca Cestari Zychar, Rodrigo Pessôa, Alberto José da Silva Duarte, Patricia Bianca Clissa, Sabri Saeed Sanabani

**Affiliations:** 1 Post-Graduation Program in Translational Medicine, Department of Medicine, Federal University of São Paulo, São Paulo 04021-001, Brazil; andrezza.ns@gmail.com (A.N.); rodrigo_pessoa1@hotmail.com (R.P.); 2 Laboratory of Pathophysiology, Butantan Institute, São Paulo 05503-900, Brazil; bianca.zychar@butantan.gov.br; 3 Laboratory of Dermatology and Immunodeficiency, Department of Dermatology LIM 56, Faculty of Medicine, University of São Paulo, São Paulo 05403-000, Brazil; alberto.duarte@hc.fm.usp.br; 4 Laboratory of Immunopathology, Butantan Institute, São Paulo 05503-900, Brazil; 5 Laboratory of Medical Investigation 03 (LIM03), Clinics Hospital, Faculty of Medicine, University of São Paulo, São Paulo 05403-000, Brazil

**Keywords:** small RNAs, jararhagin, venom

## Abstract

Small RNAs (sRNAs) and microRNAs (miRNAs) are small endogenous noncoding single-stranded RNAs that regulate gene expression in eukaryotes. Experiments in mice and humans have revealed that a typical small RNA can affect the expression of a wide range of genes, implying that small RNAs function as global regulators. Here, we used small RNA deep sequencing to investigate how jararhagin, a metalloproteinase toxin produced from the venom of *Bothrops jararaca*, affected mmu-miRNAs expression in mice 2 hours (Jar 2hrs) and 24 hours (Jar 24hrs) after injection compared to PBS control. The findings revealed that seven mmu-miRNAs were substantially differentially expressed (*p* value (*p* (Corr) cut-off 0.05, fold change ≥ 2) at 2 hrs after jararhagin exposure and that the majority of them were upregulated when compared to PBS. In contrast to these findings, a comparison of Jar 24hrs vs. PBS 24hrs demonstrated that the majority of identified mmu-miRNAs were downregulated. Furthermore, the studies demonstrated that mmu-miRNAs can target the expression of several genes involved in the MAPK signaling pathway. The steady antithetical regulation of mmu-miRNAs may correlate with the expression of genes that trigger apoptosis via MAPK in the early stages, and this effect intensifies with time. The findings expand our understanding of the effects of jararhagin on local tissue lesions at the molecular level.

## 1. Introduction

Snakebite is a neglected disease in many tropical and subtropical developing countries. According to available data, the annual global death toll from snakebite envenomation is over 125,000, with an estimated 400,000 people suffering permanent physical disabilities and over 6 million disability-adjusted life years [1]. The majority of accidents reported to the Ministry of Health in Brazil are caused by venomous snakes of the *Bothrops* genus [2,3]. Bothropic envenomations are characterized by systemic reactions such as severe blood clotting disorders, as well as serious local reactions such as edema, pain, hemorrhage, and necrosis, and there may be large tissue losses with the possibility of amputations [4]. Clinically, serum therapy is the only effective treatment for *Bothrops* envenomation; however, this approach is ineffective in reducing the fast-setting local effects induced by envenomation, implying that activation of endogenous mediators plays an important role in the local reaction [5]. Previous studies conducted by our group have shown that the proinflammatory effect of *Bothrops jararaca* venom is caused by the presence of metalloproteases in the venom [6,7].

The venom of *B. jararaca* is extremely protein rich and contains a range of enzymes, including metalloproteases, serine proteinases, phospholipases A2 (PLA2), and L-amino acid oxidases [8,9]. Several metalloproteases have been identified from the venom of *B. jararaca*, including bothropasin, HF3 [10] jararafibrase I [11], and jararhagin [12]. The best-characterized of these is the snake venom metalloproteinase jararhagin (SVMJ), which has a structure that includes the metalloproteinase domains, ECD-disintegrin domain (ECD: Glu-Cys-Asp), and cysteine-rich domain [5,12] characteristic of a PIII-type snake venom metalloproteinase (SVMP) [13]. When jararhagin is injected intradermally into mouse skin, hemorrhage occurs within minutes. This happens when basement membrane (BM) proteins such as laminin, nidogen, and type IV collagen that surround endothelial cells in capillaries breakdown. This, along with the action of hemodynamic biophysical forces, can cause a mechanical disruption of BM structure, leading to local bleeding and, in some cases, a systemic effect (bleeding) [2,14]. The proinflammatory in vivo response induced by jararhagin is characterized by an increase in the adhesion and migration of leukocytes [15] with polymorphonuclear and mononuclear cell infiltrates [16]. The proinflammatory cytokines, TNF-α, IL-6, and IL-1β released in the local tissue damage (LTD) [17], followed by mechanical hyperalgesia with the involvement of the proinflammatory cytokines TNF-α and IL-1β, and the nuclear transcription factor “NFkB” [18]. The chronic administration of jararhagin in the in vivo mouse sponge model demonstrated that this SVMP can modulate inflammatory angiogenesis, increasing inflammatory markers, such as chemokines CXCL-1 and CCL2 and cytokine TNF-α, promoting neutrophil, and macrophage activation. Jararhagin also modulated the neovascularization increasing the VEGF levels, as well as fibrogenesis markers such as TGF-β [19].

Small RNAs (sRNAs), which include microRNAs (miRNAs), are short endogenous, noncoding RNAs of 18–22 nucleotides in length that regulate gene expression at the posttranscriptional level by inhibiting translation or catalyzing gene degradation when bound to them via protein-mediated base-pairing [20]. These molecules play important roles in a variety of cellular processes, such as cell proliferation [21], differentiation [22], apoptosis [23], and inflammation [24]. The strict control of the expression of these miRNAs is critical for controlling the progression of several diseases, including cancers [25], neurological diseases [26,27], and inflammatory disorders [28,29]. As a result, we hypothesized that miRNAs might play an important role in the events involved in the pathogenesis of the jararhagin-induced tissue injury.

The goal of this study was to search for changes in sRNA levels including musculus microRNAs (mmu-miRNA) in murine gastrocnemius muscle two and twenty-four hours after intramuscular (i.m.) jararhagin injection. We also investigated whether miRNA signatures identify some common mechanisms of tissue damage using target genes and pathway analysis.

## 2. Results

### 2.1. Known sRNA Expression Profile Following Jararhagin and PBS Challenge

**Figure 1:**
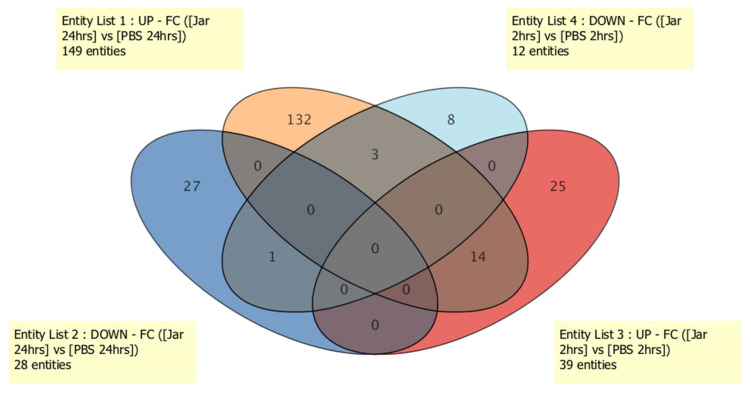
Venn diagram of known sRNA expression among groups.

Here, we aimed to determine the role of sRNAs in jararhagin- and PBS-induced innate immune responses by measuring known sRNAs, novel sRNAs and mature miRNA levels in the gastrocnemius muscle at two time points over 24 hrs. To this end, the time-dependent changes in the sRNA expression profile in RNA purified from gastrocnemius muscle at 2 hrs and 24 hrs were measured using the Illumina MiSeq massive parallel sequencing (MPS) approach. Exposure to jararhagin resulted in a general dysregulation of 302 known sRNA levels at both time points when compared to PBS (Table S1). Of the 302 known sRNAs after 2 hrs of jararhagin challenge, 19 were miRNAs (13 downregulated and 6 upregulated), 124 were snoRNAs (77 downregulated and 47 upregulated), 33 were snRNAs (13 downregulated and 20 upregulated), and 126 were tRNAs (46 downregulated and 80 upregulated). The top ten significantly downregulated known sRNAs were nine snoRNAs (Snord68, Snord91, Snora21, AF357341, Snord83, Snora69, Snora38, Snord88, and Snord71) and one tRNA (trna28) (average fold change (FC) = −2.5 × 10^7^) , whereas among the top ten significantly upregulated known sRNAs (average FC = 2.5 × 10^7^), five were tRNAs (trna107, trna994, trna699, trna110, and trna1747), four were snoRNAs (U3 GENE ID: ENSMUSG00000094705, ENSMUSG00000093842, ENSMUSG00000094480, and ENSMUSG00000096428), and one was a miRNA (mmu-mir-451a). Differential patterns of expression of the 302 known sRNAs at 24 hrs after challenge with jararhagin and PBS revealed 5 downregulated and 14 upregulated miRNAs, 23 downregulated and 101 upregulated snoRNAs, 6 downregulated and 27 upregulated snRNAs, and 27 downregulated and 99 upregulated tRNAs. Of these, the top ten significantly downregulated known sRNAs were five snoRNAs (U3, SNORA8, SNORA66, SNORD13, and SNORA65), three tRNAs (trna91, trna979, and trna969), and two miRNAs (mmu-mir-6240 and mmu-mir-10a) (average FC = −2.6 × 10^7^), whereas the top ten considerably upregulated known sRNAs were seven snoRNAs (SNORD20, SNORD44, AF357341, SNOU105B, SNORD8, SNORA57, and SNORA73a), one tRNA (trna1432), and two miRNAs (mmu-mir-3535, mmu-mir-434) (average FC = 5.5 × 10^7^). As shown in Figure 1, there were 3 commonly dysregulated known sRNAs between entity list 1 (upregulated sRNAs in Jar 24hrs vs. PBS 24hrs) and entity list 4 (downregulated sRNAs in Jar 2hrs vs. PBS 2hrs), 14 sRNAs that were upregulated in entity list 1 (Jar 24hrs vs. PBS 24hrs) compared to entity list 3 (Jar 2hrs vs. PBS 2hrs), and 1 downregulated sRNA between entity list 2 (Jar 24hrs vs. PBS 24hrs) and entity list 4 (Jar 2hrs vs. PBS 2hrs).

**Figure 2:**
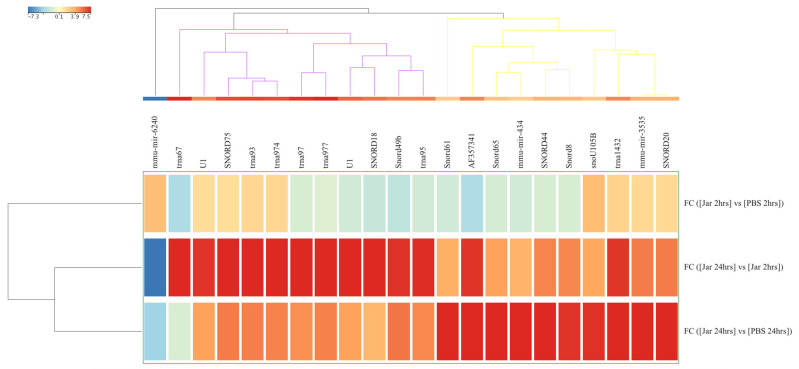
Differences in known small RNA (sRNA, *n* = 22) expression levels from gastrocnemius muscle samples obtained after 2 hrs and 24 hrs of challenge with jararhagin and PBS. The sample-clustering tree is displayed to the left and the sRNA clustering tree is above forming 3 clusters as indicated by black, purple, and yellow colors. The color scale at the top indicates the relative expression levels of sRNA across all samples. Red indicates a high expression level and blue indicates a low expression level. Each column represents one known sRNA, and each row represents one sample.

The expression analysis was repeated with a stringent statistical parameter of FDR *p* value of <0.05 and FC ≥ 5 and revealed 22 significantly dysregulated sRNAs. A heatmap of the hierarchical clustering of these sRNAs is shown in Figure 2. 

**Figure 3:**
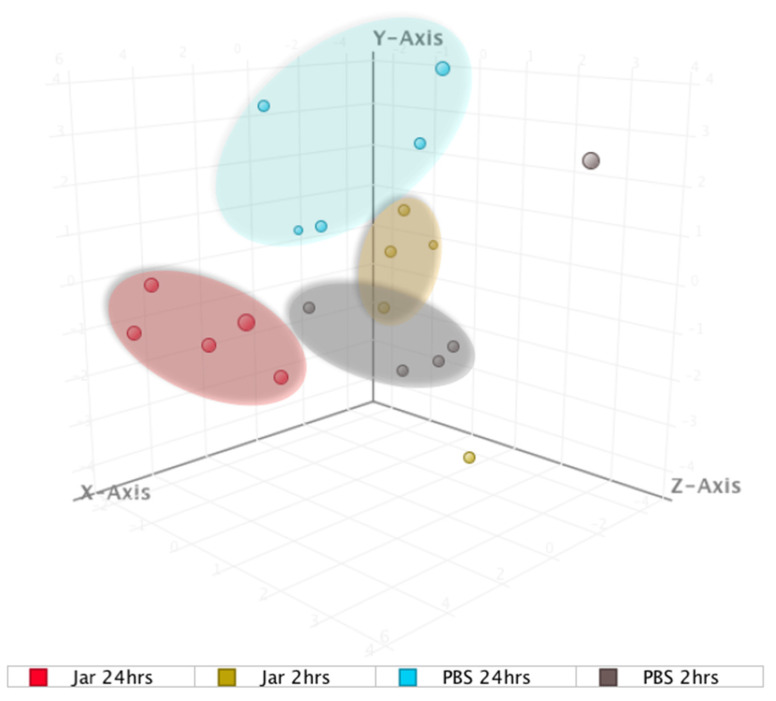
Principal component analysis (PCA) plot showing distances among the four groups based on the profile of the 22 significantly expressed known small RNAs. The shaded circles highlighted (yellow, blue, gray, red) refer to the clusters formed from small RNA molecular data.

Principal component analysis (PCA) was performed using the 22 strongly dysregulated sRNAs to visualize how closely related the four groups were regarding their sRNA expression patterns (Figure 3). The results showed Jar 2hrs and Jar 24hrs as clearly separated clusters, while Jar 2hrs samples minimally overlapped with the PBS group. Overall, the results showed that the response was time-dependent and that sRNAs peaked at 24 hrs.

### 2.2. Novel sRNA Expression Profile Following Jararhagin and PBS Challenge

**Figure 4:**
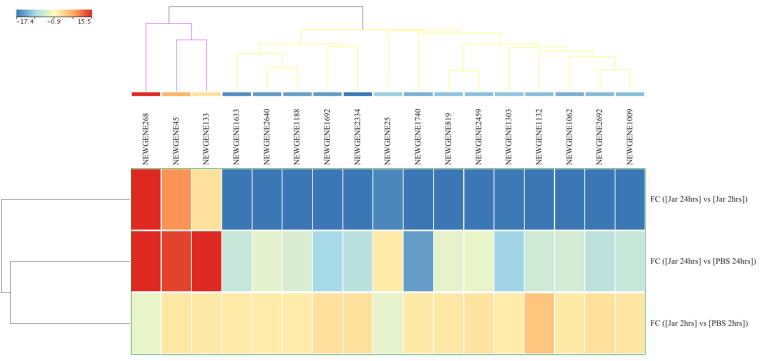
Differences in novel small RNA (sRNA, *n* = 17) expression levels from gastrocnemius muscle samples obtained after 2 and 24 hrs of challenge with jararhagin and PBS. The sample-clustering tree is displayed to the left, and the sRNA clustering tree is above forming two clusters as indicated by purple and yellow colors. The color scale at the top indicates the relative expression levels of sRNA across all samples Red indicates a high expression level and blue indicates a low expression level. Each column represents one known sRNA and each row represents one sample.

A total of 3067 novel sRNAs were detected in all samples. Of these, 1851 (60.4%) were snoRNAs and 1216 (39.6%) were unknown novel sRNAs. Only 17 novel genes reached FDR significant value (*p* (Corr) cutoff < 0.05) and FC ≥ 10 during the comparison of the jararhagin and PBS groups at two time points. A comparison of the expression levels of these genes between Jar 2hrs vs. PBS 2hrs showed two unknown downregulated and 15 upregulated genes (8 snoRNAs and 7 unknown RNAs). The most significantly up- and downregulated genes in this group were unknown sRNAs designated as NEWGENE1132 (FC = 3.9 × 10^7^) and NEWGENE25 (FC = −1.7 × 10^7^), respectively (Table S2). Analysis of Jar 24hrs vs. PBS 24hrs demonstrated 13 downregulated genes, of which eight were snoRNAs and five were unknown identities. Within this group, the snoRNA (NEWGENE1303) and unknown sRNA (NEWGENE25) were the most significantly downregulated (FC = −5.7 × 10^7^) and upregulated (FC = 1.3 × 10^7^) genes, respectively. The comparison between Jar 24hrs and Jar 2hrs revealed 14 downregulated genes, and 8 of them were snoRNAs. The top downregulated gene was snoRNA (NEWGENE2334, FC = −1.8 × 10^7^). Three unknown genes within this group were upregulated, among which the gene designated as NEWGENE133 was the most upregulated novel gene in Jar 24hrs compared to Jar 2hrs (Figure 4).

### 2.3. Mature miRNA Expression Profile Following Jararhagin and PBS Challenge

**Figure 5:**
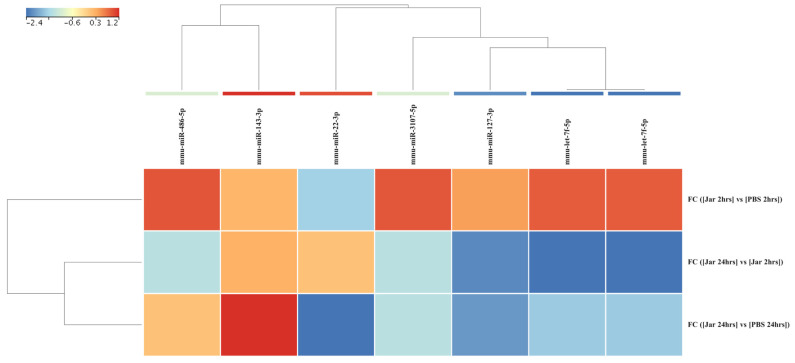
Differences in mature mmu-miRNA (*n* = 7) expression levels from gastrocnemius muscle samples obtained after 2 and 24 hrs of challenge with jararhagin and PBS. The sample clustering tree is displayed to the left and the sRNA clustering tree is above. The color scale at the top indicates the relative expression levels of sRNA across all samples. Red indicates a high expression level and blue indicates a low expression level. Each column represents one known sRNA, and each row represents one sample.

**Figure 6:**
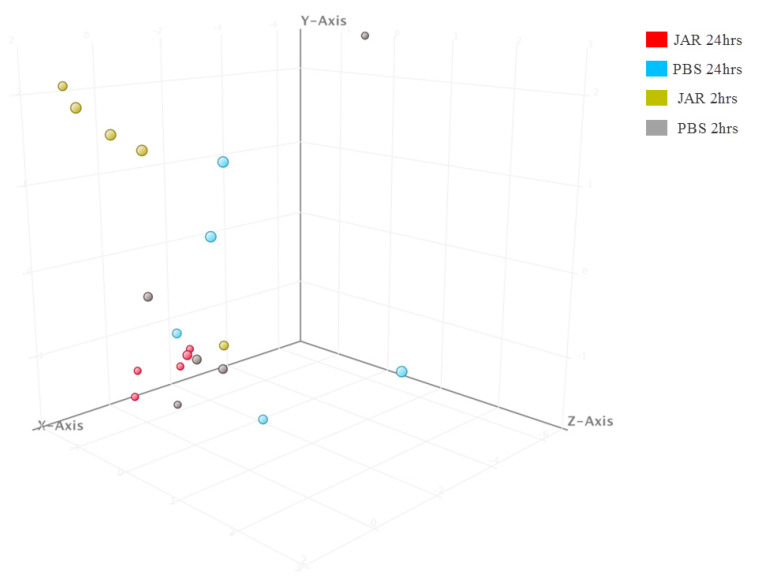
Principal component analysis (PCA) plot showing distances among the four groups based on the profile of the seven significantly expressed mmu-miRNAs.

Investigation of mature mmu-miRNA among the jararhagin and phosphate-buffered saline (PBS) challenged groups at the two time points revealed seven significantly dysregulated (*p* value (*p* (Corr) cutoff < 0.05, FC ≥ 2) mmu-miRNAs (Table S3). Most of these mmu-miRNAs were significantly upregulated in Jar 2hrs vs. PBS 2hrs. mmu-miR-486-5p was the most upregulated gene (FC = 2.3 × 10^7^) followed by mmu-let-7f-5p (FC = 2.2 × 10^7^). In contrast to the results of Jar 2hrs vs. PBS 2hrs, the analysis of Jar 24hrs vs. PBS 24hrs revealed that the majority of detected mmu-miRNAs were downregulated. mmu-miR-22-3p and mmu-miR-127-3p were the most downregulated miRNAs (average FC = −2.2 × 10^7^). Additionally, mmu-miR-22-3p expression was downregulated in a time-dependent manner ranging from FC = −1.3 × 10^7^ at 2hrs to FC = −2.4 × 10^7^ at 24 hrs compared to the PBS control group. The unsupervised hierarchical cluster analysis of the seven differentially expressed mature mmu-miRNAs of the three groups is displayed in Figure 5. In the PCA plot using the seven dysregulated mmu-miRNAs, the Jar 2hrs and Jar 24hrs samples clustered into two distinct groups (Figure 6). Furthermore, the Jar 24hrs samples showed a higher degree of heterogeneity and minimal overlap with both the Jar 2hrs, PBS 2hrs, and/or 24 hrs samples. Overall, the PCA plot showed that mmu-miRNAs can separate gastrocnemius muscle challenged with jararhagin at 24 hrs from samples challenged at 2hrs.

### 2.4. Target Genes, KEGG Pathway, and GO Enrichment Analysis

**Figure 7:**
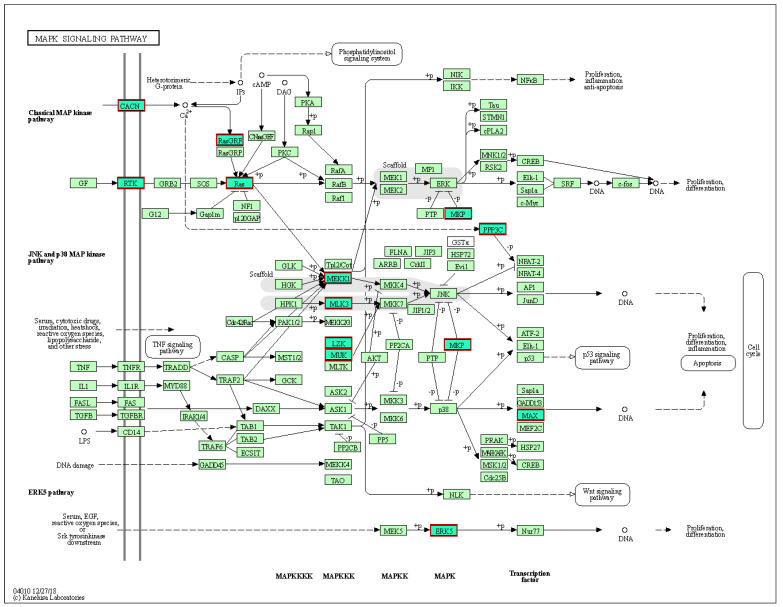
KEGG pathway for MAPK signaling pathway. The output of miRWalk analysis demonstrating the MAPK signaling pathway significantly enriched for significantly dysregulated mature mmu-miRNAs. Red boxes shaded in green denote predicted target genes. Gray shades indicate scaffolding.

To investigate the possible functions of the seven mmu-miRNAs in the immune response toward jararhagin, the miRWalk v3 online tool was used to predict target genes and pathways potentially influenced by these mmu-miRNAs. In total, 175 unique genes were predicted by at least three prediction tools for four miRNAs, namely mmu-miR-22-3p, 127-3p, 143-3p, and mmu-let-7f-5p (Table S4). mmu-miR-22-3p exhibited 56 target genes, whereas mmu-miR-127-3p exhibited three target genes (Slc12a4, Spock2, Septin7), mmu-miR-143-3p exhibited 33 target genes, and mmu-let-7f-5p exhibited 83 target genes. There were eight KEGG pathways enriched with genes targeted by the four miRNAs, among which the most statistically significant pathway was the MAPK signaling pathway (mmu04010, FDR corrected *p*-value = 3.8 × 10^−3^) (Table S5, Figure 7). Subsequently, GO annotations were performed by the predicted target genes (Table S5). The GOCC enrichment results showed that norepinephrine epinephrine mediated vasodilation involved in regulation of systemic arterial blood pressure (GO:0002025) and activin receptor signaling pathway (GO:0032924) were the top significantly enriched processes (FDR corrected *p* value of ≤0.05). In the CC category, the target genes were significantly enriched in various processes including chromatin (GO:0000785), T-tubule (GO:0030315), nuclear envelope (GO:0005635), and Schaffer collateral–CA1 synapse (GO:0098685). In the MF category, the target genes were significantly and mainly enriched in guanyl-nucleotide exchange factor activity (GO:0005085) and DNA-binding transcription factor activity (GO:0003700).

## 3. Discussion

Jararhagin triggers the activation of innate immune cells and causes a strong proinflammatory response characterized by marked recruitment and accumulation of leukocytes at the inflammation site and induction of proinflammatory cytokines and apoptotic macrophages [16,17,30,31]. It is believed that alterations in molecular processes are at least partially mediate these events [32,33]. Several studies have demonstrated that snake venom to exhibits geographic and ontogenetic variation [33,34,35]. To date, no reports are available on sRNA sequencing profile of injured tissue as a result of snake bite. Nevertheless, numerous studies have demonstrated the roles of miRNAs in many biological events, including cell death, differentiation, proliferation, and cell growth [36,37]. Here, we generated wide genome small RNA sequencing data from mouse gastrocnemius muscle challenged with jararhagin at two time points to investigate the temporal role of the sRNAome in jararhagin injured tissue. Our study reveals that the expression levels of miRNAs are altered when local tissues are exposed to jararhagin, indicating a role for miRNAs in the cellular response to SVMP in these tissues. This may indicate that local tissues employ the activation and transcription of miRNAs, as well as gene translation, two hours after exposure. It is also possible that the synthesis of miRNAs begins early on in the inflammatory response of these local lesions to jararhagin. Furthermore, we show that miRNA temporal expression alterations in response to jararhagin are different in local lesions. Two hours after challenge, we identified the dysregulation of 302 sRNAs. Among them, 18 sRNA transcripts were dysregulated at 24 hrs after challenge (Figure 1). Our results also demonstrate that mum-miRNAs, particularly mmu-miR-22-3p, 127-3p, 143-3p, and mmu-let-7f-5p, are actively dysregulated at both time points. We also found that the four altered mmu-miRNAs converge on the MAPK signaling pathway. Of note, the MAPK signaling pathway has been shown to mediate the activation of intracellular PLA2 in physiological and inflammatory contexts [38,39]. Thus, we assume the MAPK signaling pathway is an underlying pathogenic mechanism in jararhagin injured tissue.

Here, we show that the tissue levels of mum-miRNA-22-3p in mice challenged with jararhagin were considerably lowered at 2hrs and continued to decline at 24 hrs. Although a link between mum-miRNA-22-3p and tissue damage caused by the *B. jararaca* venom has not been previously reported, miR-22-3p in humans has been linked to other chronic inflammatory diseases. For example, miR-22-3p has been reported to be elevated in B cells from systemic lupus erythematosus subjects compared to healthy human subjects [40]. Additionally, Pei et al. [41] recently reported that miR-22-3p levels are elevated in peripheral CD4+ T cells in inflammatory bowel disease. Inhibition of miR-22 was linked to increased astrogliosis [42], and its overexpression has been shown to protect against brain injury in animal models [43]. In a study by Jovicic et al. [43], miR-22 was also shown to reduce apoptosis, as evidenced by its ability to inhibit effector caspase activation in Huntington’s disease. To date, no study has evaluated the role of miRNA-22-3p in the affected tissue following *B. jararaca* bites. It is tempting to speculate that the effect of the severe tissue injury is mediated, at least in part, by targeting the proapoptotic gene MAPK. Targeting broad antiapoptotic and MAPK pathways, as well as evidence that miR-22 is downregulated in injured tissue, makes miR-22 an especially intriguing approach for treating tissue damage and inflammation following *B. jararaca* bites.

Regarding mmu-miR-127-3p, it was recently demonstrated that this miR in mouse skin wounds triggered a prolonged cell cycle arrest with unique molecular hallmarks of senescence, including activation of senescence-associated β-galactosidase, increases in p53 and p21 levels, inhibition of lamin B1 and proliferation factors, and the production of senescence-associated inflammatory and extracellular matrix remodeling components [44]. Several studies have demonstrated that miR-127-3p plays a major antitumor role in various cancers [45,46,47]. The results recently reported by DU et al. [48] revealed that the MAPK4 gene was a new candidate gene and might be a target gene for miR-127-3p in ovarian cancer. In our study, we showed that mmu-miR-127-3p was downregulated after challenge with jararhagin at both time points. Although speculative, a possible scenario is that the downregulation of mmu-miR-127-3p resulted in the upregulation of MAPK pathways, as MAPK is a direct target for mmu-miR-127.

Although we evaluated the expression of sRNA, including mature mm-miRNA, in local lesions, a prospective validation study is required to establish the levels or expression of their predicted target genes. Another limitation is that we restricted our study and interpretation mostly to miRNAs.

## 4. Conclusions

In conclusion, our study provides additional evidence that jararhagin promotes a time-dependent change in the expression levels of miRNAs, suggesting its role in the cellular response to tissue injury. In addition, we show evidence that, in response to venom exposure, distinct miRNAs are downregulated at distinct time points in local lesions. Studies addressing the mechanisms underlying the steady reduction in the miRNA described in this study are warranted. Understanding the involvement of microRNAs in tissue injury caused by SVMP jararhagin and identifying their specific targets in local lesions warrants additional investigation that may lead to the development of novel therapeutic approaches for tissue injury.

## 5. Materials and Methods

### 5.1. Experimental Design

Males Swiss mice weighing 20–25 g were obtained from the Instituto Butantan’s housing facility, São Paulo, Brazil. Before the experiments, the animals were kept 48 hrs in a 12:12 hrs light:dark cycle and had access to food and water ad libitum. All experimental procedures were carried out in accordance with the ethical standards proposed by the International Society of Toxinology and the Brazilian College of Experimental Animals and were approved by the Butantan Institute’s Ethical Committee, São Paulo, Brazil, for the Use of Animals (CEAU n 2614060420, 18 September 2020). To determine the inflammatory response induced by jararhagin, ten mice were separated into two groups of five. Both groups were challenged with 1 µg of jararhagin i.m. into the gastrocnemius muscle in the right paw and PBS in the left paw. Mice were sacrificed after 2 hrs and 24 hrs, and muscles from the right paw (designated Jar 2hrs and 24 hrs) and left paw (designated PBS 2hrs and 24 hrs) were dissected and minced, and cells were harvested in TRIzol and stored at −80 °C until use.

### 5.2. Jararhagin Purification and Depyrogenation

Jararhagin was purified from *B. jararaca* venom by hydrophobic interaction (Hi Trap Phenyl FF Low Sub 5mL-Cytiva) and anion exchange chromatography (Mono QTM 5/50 GL-Cytiva) as previously described by Paine et al. {Paine, 1992 #12} and tested for purity by SDS-PAGE and for biological activity by hemorrhagic lesion diameter after 2 hrs following intradermal injections of jararhagin in a total volume of 50 uL {Theakston, 1983 #54}. To remove remaining lipopolysaccharide (LPS) contaminations from purified jararhagin, this toxin was submitted to 1 cycle of treatment by Triton X-114 as previously described {Aida, 1990 #55}. The presence of LPS in treated samples was evaluated by Limulus Amebocyte Lysate test (LAL-Charlys River, Wilmington, MA, USA). The hemorrhagic activity of Triton-treated jararhagin was measured in the mouse skin jararhagin LPS-free sample was used for all in vivo experiments {Theakston, 1983 #54}.

### 5.3. RNA Extraction

Each gastrocnemius muscle sample was separately homogenized and RNA was extracted using the miRCURY RNA Isolation kit (Exqon, Vedbæk, Denmark) according to the manufacturer’s instructions. The resulting RNA was eluted with RNase-free water and stored at −80 °C until further use. sRNA quantities were measured using a Qubit 2.0 fluorometer with a microRNA Assay Kit (Thermo Fisher Scientific, Inc., Waltham, MA, USA).

### 5.4. Library Construction

For each sample in both groups, sRNA libraries were prepared with the TruSeq Small RNA Sample Preparation Kit (Illumina, San Diego, CA, USA) per the manufacturer’s instructions and a previously published protocol [49,50]. A total library pool of 4 nM was prepared using a MiSeq Reagent Kit v3 150 cycle followed by sequencing on a MiSeq system (Illumina, San Diego, CA, USA). The libraries were sequenced on a 150-SE run on the MiSeq with a 36-base single-end protocol [51]. After trimming adapter sequences and sequence quality testing, each library’s raw data were aligned to the human reference genome (hg19), combined into an expression matrix and processed with Strand NGS version 3.1 (Strand Life Science, Bangalore, India). The distributions of the sRNA data in each clinical condition were conducted according to the quantile normalization algorithm, with a baseline transformation set to the median of all samples. Only miRNAs with more than ten copies were considered for subsequent analysis. Mmu-miRNAs with a fold-change (FC) ≥2 were considered to be differentially expressed. All sequence data described here are available in the online Zenodo repository: https://doi.org/10.5281/zenodo.6599508 (accessing date 31 May 2022).

### 5.5. Functional Annotation and Pathway Analysis of miRNA Target Genes

The target genes from differentially expressed mature mmu-miRNAs of were predicted by the miRWalk 3.0 algorithm (Mannheim, Germany). After obtaining a list of putative and experimentally validated targets relative to each mmu-miRNA, we further scanned these targets and analyzed them for Gene Ontology (GO) enrichment terms and Kyoto Encyclopedia of Genes and Genomes (KEGG) pathway classification. The target genes for the significantly dysregulated miRNA were interrogated for significant well-curated signaling pathways obtained from Reactome using miRWalk v.3 (Mannheim, Germany) (March 2020 update) sorted by *p* value ranking <0.05 using Benjamini–Hochberg multiple testing correction to control the false discovery rate (FDR).

### 5.6. Constructing Regulatory Network between miRNAs and Their Targets

The posttranscriptional gene regulatory network is defined as a directed and bipartite network in which the expressions levels of miRNA target gene interacting pairs are reversely correlated. The analysis of the network for the interaction of miRNA-messenger RNA (mRNA) putative targets was performed using the miRWalk network algorithm [52].

## Data Availability

All sequence data described here are available in the online Zenodo repository at https://doi.org/10.5281/zenodo.6599508.

## Supplementary Materials

The following supporting information can be downloaded: https://www.mdpi.com/article/10.3390/toxins14070472/s1, Table S1: known sRNAs differentially expressed in all groups, Table S2: Novel small RNAs identified in this study; Table S3: Mature miRNAs differentially expressed in all groups, Table S4: Target genes identified in the miRWalk for the four differentially expressed mature mmu-miRNAs, Table S5: Enriched pathways and GO terms in gastrocnemius muscle samples injected with jararhagin. The results were obtained from the target genes identified in the miRWalk for the four differentially expressed mmu-miRNAs.
